# Studies on p53, BAX and Bcl-2 protein expression and microsatellite instability in stage III (UICC) colon cancer treated by adjuvant chemotherapy: major prognostic impact of proapoptotic BAX

**DOI:** 10.1038/sj.bjc.6603728

**Published:** 2007-04-10

**Authors:** O Nehls, T Okech, C-J Hsieh, T Enzinger, M Sarbia, F Borchard, H-H Gruenagel, V Gaco, H G Hass, H T Arkenau, J T Hartmann, R Porschen, M Gregor, B Klump

**Affiliations:** 1 Department of Internal Medicine I, University Hospital, Tübingen, Germany; 2 Department of Internal Medicine II, University Hospital, Tübingen, Germany; 3 Institute of Pathology, Sana Klinikum Lichtenberg/Unfallkrankenhaus, Berlin, Germany; 4 Department of Pathology, Aschaffenburg, Germany; 5 Department of Surgery, Evangelic Hospital, Düsseldorf, Germany; 6 Clinic of Internal Medicine, Central Hospital Bremen Ost, Bremen, Germany

**Keywords:** adjuvant chemotherapy, BAT26, BAX, Bcl-2, colon carcinoma, microsatellite instability, p53/BAX pathway, prognosis

## Abstract

We evaluated the expression patterns of proapoptotic BAX, antiapoptotic Bcl-2 and p53, the proposed upstream effector of these molecules, as potential prognostic markers in UICC stage III colon cancer by immunohistochemical staining. To identify high-frequency microsatellite instability (MSI+) individuals, we performed single-strand conformation polymorphism-based analysis for BAT26. A total of 188 patients who had received 5-fluorouracil (5-FU)-based adjuvant chemotherapy (5-FU/folinic acid or 5-FU/levamisole) were enrolled. Median follow-up was 84.5 months. We found that BAX, Bcl-2 and p53 protein expressions were high or positive in 59, 70 and 50% of 188 cases, respectively. MSI+ tumours were detected in 9% of 174 evaluable patients. BAX or Bcl-2 was correlated with a higher degree of differentiation or left-sided tumours (*P*=0.01 or *P*=0.03, respectively); MSI was correlated with right-sided tumours (*P*<0.0001). In contrast to p53, Bcl-2, or MSI, low BAX, advanced pN category, low grade of differentiation and treatment with 5-FU/levamisole were univariately associated with poorer disease-free survival (DFS) (*P*=0.0005, *P*=0.001, *P*=0.005 and *P*=0.01, respectively) and poorer overall survival (OS) (*P*=0.002, *P*=0.0001, *P*=0.003 and *P*=0.02, respectively). Besides pN category and treatment arm, BAX was an independent variable related to both OS and DFS (*P*=0.003 and *P*=0.001, respectively). In both univariate and multivariate analysis, the p53−/BAX high in comparison with the p53+/BAX high subset conferred a significantly improved DFS (*P*=0.03 and *P*=0.03, respectively) as well as a marginally improved OS (*P*=0.07 and *P*=0.08, respectively). BAX protein expression may be of central significance for clinical outcome to 5-FU-based adjuvant chemotherapy in stage III colon cancer, and bivariate analysis of p53/BAX possibly may provide further prognostic evidence.

In patients with colon cancer, the extent of tumour stage, defined by the depth of invasion, involvement of lymph nodes and metastatic spread to distant organs, still confers the most useful prognostic information. However, while stages I and IV (UICC) have a uniform favourable and dismal prognosis, respectively, the 5-year survival rates reported for patients with stage II colorectal cancer vary between 62 and 70%, and between 27 and 60% for patients with stage III disease (Macdonald and Astrow, 2001; Greene *et al*, 2002). Although the benefit of adjuvant chemotherapy has clearly been demonstrated for stage III colon cancer, only 5–15% of these patients will benefit from this modality; consequently, 85–95% of these patients undergoing adjuvant chemotherapy will be overtreated (Mamounas *et al*, 1999). Therefore, novel markers that pretherapeutically identify stage III patients likely benefiting from adjuvant approaches are urgently needed.

Colon carcinogenesis has been shown to be a multi-step process of both histo-morphological and molecular alterations. Singular stages of neoplastic transformation are associated with either the inactivation of tumour suppressor or the activation of oncogenes (Fearon and Vogelstein, 1990). An alternative pathway of tumorigenesis is characterised by a defective DNA mismatch repair system and the rapid accumulation of somatic mutations (Perucho, 1996). As a consequence, the epithelial homeostasis is disrupted by an imbalance of apoptotic and mitotic processes. While accelerated proliferation increases the occurrence of genetically damaged cells, the inhibition of apoptosis is believed to confer a growth advantage and to prevent the elimination of genetically altered cells. Both mechanisms are thought to contribute to the initiation and progression of malignancies (Kerr *et al*, 1972; Williams and Smith, 1993).

In the search for better prognostic information, genes or proteins involved in these processes represent natural candidate markers, and genetic alterations in either tumour suppressor or oncogenes have been reported to be associated with a worse prognosis in colon cancer. However, in regard to the prognostic information on the majority of suggested candidate markers, results have been controversial and so far no molecular alteration has changed clinical practice outside research programmes.

The Bcl-2 family of proteins is intimately involved in the regulation of apoptosis. While Bcl-2 and Bcl-x represent important inhibitors of apoptosis, the BAX subfamily consisting of BAX, Bak as well as the BH3-only subfamily induces programmed cell death (Yang and Korsmeyer, 1996; Reed, 1997, 1998). This is also true for apoptosis induced by cytotoxic treatment (Wagener *et al*, 1996). Among the members of the Bcl-2 family, BAX is suggested to be a key regulator of the apoptotic machinery, transcriptionally activated by upstream molecules, such as the tumour suppressor p53 (Miyashita and Reed, 1995). However, BAX-induced apoptosis through p53-independent mechanisms in response to anticancer agents has been found in colorectal cell line studies as well (Ravi and Bedi, 2002).

On the other hand, it was proposed that the regulation of the cellular suicide programme in response to cellular stress depends on the dynamic interaction between Bcl-2 and BAX. Thus, higher relative BAX levels might result in apoptotic cell death, whereas higher relative Bcl-2 levels might inhibit this programme in response to genotoxic stress (Oltvai *et al*, 1994). The prognostic information of BAX protein expression has been tested for a variety of malignancies, and a recent study reported a significant worse prognosis of those patients with colorectal cancer whose liver metastasis showed low proapoptotic BAX expression (Sturm *et al*, 1999). However, data regarding the prognostic value of Bcl-2 or p53 expression in colorectal cancer are contradictory, as recently reviewed by our group and others (Grizzle *et al*, 2002; Klump *et al*, 2004).

In stage III colorectal carcinoma, patients with high-frequency microsatellite instability (MSI+) lesions have been shown to display a different prognosis than those with microsatellite-competent or low-frequency microsatellite instability (MSI−) neoplasms. Recent evidence suggests that stage III patients whose tumours are categorised as MSI+ may not benefit from 5-fluorouracil (5-FU)-based adjuvant treatment (Ribic *et al*, 2003), whereas others have found contradictory results (Elsaleh *et al*, 2001). BAT26, a mononucleotide microsatellite marker, has been demonstrated to be sufficient to screen for high-frequency MSI in colorectal cancer of Caucasian populations with a sensitivity of 97% and more (Hoang *et al*, 1997; Zhou *et al*, 1998; Loukola *et al*, 2001).

Possibly, evaluation of the interplay between different mediators instead of performing monogenic studies may help to provide more reliable molecular information to predict clinical outcome in colorectal cancer.

Accordingly, this study sought to further identify the predictive and/or prognostic significance of BAX, Bcl-2 and p53 expression and BAT26 alone, as well as the role of BAX combined with Bcl-2 or p53 or BAT26 in UICC stage III colon cancer. Since adjuvant chemotherapy represents the standard of care in stage III colon carcinoma, the influence of the above-mentioned markers on the outcome of adjuvant 5-FU-based chemotherapy was to be tested.

## MATERIALS AND METHODS

### Patients and materials

A total of 188 patients with histologically confirmed diagnosis of stage III colon cancer (UICC) were included. All patients were entered into the prospective study of the Study Group Gastrointestinal Oncology North-Rhine Westphalia between December 1991 and December 1994. In this clinical trial, a total of 680 eligible patients were randomly assigned to receive potentially curative surgical resection followed by adjuvant chemotherapy with 5-FU combined with leucovorin (arm A) or levamisole (arm B) as previously described (Porschen *et al*, 2001; Arkenau *et al*, 2003).

As it was not an obligate requirement of the protocol to send the tissue samples, archival resected specimens were available from only 204 patients. Of these, 15 patients were excluded, one of them owing to insufficient paraffin material and 14 patients because they did not receive adjuvant chemotherapy at all. Analysis for MSI status was performed on only 174 of the 188 samples because of PCR failure due to insufficient tumour DNA.

The age of patients ranged from 34 to 80 years with a median age of 62 years. We defined right-sided tumours as those arising proximal to the splenic flexure, and left-sided as those originating distal to Cannon's point. The median follow-up was 84.5 months (range 2.4–117.4 months). Further details of the study population and tumour characteristics are given in Table 1A and 1B.

The German multicentric trial was approved by the Ethical Committee of the Faculty of Medicine of the Heinrich-Heine-University of Düsseldorf.

### Histopathology

After surgery, tumour specimens, resection margins and lymph nodes were formalin fixed and paraffin embedded. Haematoxylin- and eosin-stained sections were reviewed to establish the pathological diagnosis and stage according to the tumour node metastasis classification of the UICC (Sobin and Wittekind, 1997) and the World Health Organization grading system (Jass and Sobin, 1989) (FB, MS).

### Clinical follow-up

Follow-up was performed in accordance with a standardised scheme established within the clinical trial's protocol (Arkenau *et al*, 2003). Follow-up forms were sent to participating centres for each patient on a quarterly basis for the first 2 years, on a semiannual basis for the next 3 years and yearly thereafter. Examinations included history, physical examination, blood tests, CEA determinations (optionally) and abdominal ultrasound every 3 months during the first 2 years and every 6 months thereafter. Colonoscopy and chest radiography were recommended every 6 months during the first 2 postoperative years and then once per year. Abdominal computed tomography was performed 3 months after surgery and repeated every 12 months.

Patients and treating physicians were alerted before the follow-up date to improve compliance. Patients were classified as having no evidence of disease or having evidence of relapse. Relapse was defined as local recurrence, the manifestation of a secondary metachronous tumour or development of distant metastasis.

### Immunohistochemistry

Paraffin sections (5 *μ*m) were deparaffinised and rehydrated through graded alcohols. Endogenous peroxidase activity was blocked by incubation with 0.3% hydrogen peroxide for 10 min. Antigen retrieval was performed by fractioned (3 × 5 min) microwave treatment in citrate buffer (pH 6.0). Subsequently, sections were covered for 30 min at room temperature with normal horse serum (for p53), normal swine serum (for BAX) and normal cow serum (for Bcl-2) to block nonspecific background staining before incubation with primary antibodies. Primary antibodies were the monoclonal antibody DO-1 for p53 (Oncogene Science, Cambridge, MA, USA; dilution 1 : 50 for 1 h), the rabbit anti-human antibody for BAX (N20, Santa Cruz Biotechnology Inc., Santa Cruz, CA, USA; dilution 1 : 200 for 1 h30) and the mouse anti-human monoclonal antibody for Bcl-2 (Dako Diagnostika, Hamburg, Germany; dilution 1 : 30 overnight). Slides were incubated with diluted primary antibodies for p53 and BAX at room temperature, whereas incubation for Bcl-2 was performed at 4°C. Subsequently, slides were covered with appropriate biotinylated secondary antibody at room temperature and an avidin-biotinylated peroxidase complex (Vectastain ABC Kit, Vector Labs, Burlingame, CA, USA) for 30 min each. Sections were then developed using 3,3′-diaminobenzidine and counterstained with haemalaun solution and mounted using aqueous medium. Slides were washed twice between incubations with Tris-buffered saline (pH 7.4). For each antibody, a known positive colon cancer specimen served as positive control. Negative control slides were processed with each slide run excluding the primary antibody but with inclusion of all other steps of the procedure. Randomly repeated slide runs showed the same staining properties, depicting the consistency of the staining.

### Quantification of immunostaining

Expression patterns were independently determined by two investigators in a semiquantitative manner by light microscopy considering whole tumour specimen slides blind to clinicopathological variables and outcome, respectively. In case of discrepant results, a final consensual decision was made.

Immunoreactivity for p53 (nuclear staining) and Bcl-2 (cytoplasmic staining) was categorised in accordance with the percentage of tumour cells stained as described previously: immunonegative, ⩽5%; immunopositive, >5% staining.

BAX staining index (BSI) was established based on information on both staining intensity and the percentage of positive cells within a tumour. Five categories were defined for the percentage of positive cells (1, 0%; 2, >0–60%; 3, >60–80%; 4, >80–99%; and 5, 100%). Four categories were defined for staining intensity (1, not detectable; 2, weak; 3, moderate; and 4, strong). The BSI was defined as the product of score points found for both intensity and percentage of positive cells (i.e. 1–20 points achievable). For further univariate and multivariate analyses, samples were dichotomised according to the median BSI value slightly modified as described previously (Nehls *et al*, 2005) as follows: low BAX expression, BSI <12, and high BAX expression, BSI⩾12.

### Molecular analysis

For MSI analysis, tumour DNA was extracted from formalin-fixed paraffin-embedded surgical resection specimens. Regions with invasive cancer were identified on a reference H&E-stained sample and then microdissected with a surgical scalpel blade. The specimen was deparaffinised and then the extracted DNA was amplified by PCR with the use of the BAT26 mononucleotide microsatellite marker as described by Hoang *et al* (1997). BAT26-amplified PCR products were run on nondenaturing polyacrylamide gels and stained with ethidium bromide. Samples were dichotomised into presence (MSI+) or absence of MSI (MSI−) including low-frequency MSI cases or microsatellite stable lesions.

### Statistical analysis

The association of immunoreactivity and other established or suggested factors of prognostic impact in colon cancer (i.e. age, sex, pT category, pN category, tumour differentiation, tumour localisation and treatment arm) was analysed as dichotomised variables using the *χ*^2^ test. Primary statistical outcomes were disease-specific overall survival and disease-free survival (OS/DFS) measured from the date of surgery; OS and DFS were estimated by Kaplan–Meier curves and curves were compared by means of the log-rank test. Time to relapse and to death were analysed using the Cox proportional hazards model for univariate and multivariate analysis in a stepwise manner. Potential prognostic factors were entered into a stepwise regression model from which significance levels were determined. In addition, the hazard ratios between prognostic groups and their 95% confidence intervals were computed. Statistical significance was defined as a two-sided *P*<0.05. Statistical analysis was performed using the SPSS statistical software program (SPSS Inc., Chicago, IL, USA).

## RESULTS

### Clinical course of patients

Median duration of follow-up after surgery was 84.5 months or 6.6 years, with a range of 2.4–117.4 months. A relapse was diagnosed in 38.3% of patients. Five-year survival was 69.9%.

### Immunohistochemistry

**Figure 1:**
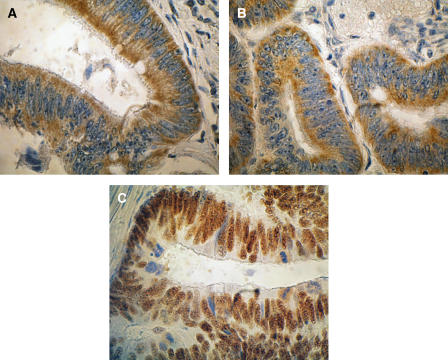
Light microscope images (× 40) of immunohistochemistry of representative colon cancer samples classified as BSI high (BAX was quantitatively detected in all positive stained cells and BAX staining intensity was classified as strong) (**A**), Bcl-2 immunopositive (>5% cytoplasmic staining) (**B**) and p53 immunopositive (>5% nuclear staining) (**C**).

Cytoplasmic BSI was classified as high (Figure 1A) and low in 111 (59%) and 77 (41%) tumours, respectively. Cytoplasmic Bcl-2 expression was positive (Figure 1B) and negative in 131 (70%) and 57 (30%) samples, and nuclear p53 immunoreactivity was categorised in 94 (50%) tumours as positive (Figure 1C) and negative, respectively.

In seven specimens (3.7%), no staining of BAX was detectable. Any immunoreactivity for BAX was quantitatively detected in 181 (96.3%) tumour specimens: in 15 (8%) between 1 and 60% of cells, in 33 (17.6%) between 61 and 80%, in 39 (20.7%) between 81 and 99%, and in 94 (50%) in all positive stained cells. Qualitatively, BAX staining intensity was weak in 52 (27.7%) of the samples, moderate in 72 (38.3%) and strong in 57 (30.3%).

Bcl-2 was detected in 57 (30%) of the samples in 5% of cells or less, in 34 (18%) between >5 and 20%, in 11 (6%) between >20 and 50%, and in 86 (46%) in more than 50%.

p53 was found in 94 (50%) of the tumours in 5% of cells or less, in 25 (13%) in >5–25%, in 3 (2%) between >25 and 50%, and in 66 (35%) in more than 50%.

BAX, Bcl-2 and p53 immunostainings were strong in all normal colon tissue samples, which served as controls.

### Molecular analysis

Of the 174 evaluable tumour specimens tested for MSI status, 16 (9%) demonstrated MSI+ and 158 tumours (91%) were categorised as MSI−.

### Univariate analysis of clinicopathological and genetic correlations

By univariate analyses, BAX (BSI), Bcl-2 and p53 immunostainings were compared with treatment arm and various tumour characteristics (sex, age, pT category, pN category, grade of differentiation (G category) or localisation). In contrast to other tumour parameters and treatment arm, a significant association of G category and BSI was detected with tumours of lower differentiation showing a decreased level of BAX protein expression (*P*=0.01). Furthermore, a significant correlation of tumour location and Bcl-2 expression or the MSI phenotype was found with left-sided tumours revealing higher Bcl-2 expression levels (*P*=0.03) or with right-sided neoplasms showing an association with MSI+ cases (*P*<0.0001) (Table 1A and 1B).

High BSI was positively correlated to Bcl-2 immunopositivity (*P*=0.002; *χ*^2^ test), whereas no significant association was found among p53 and BSI or Bcl-2 (*P*=0.5 and *P*=0.9, respectively; *χ*^2^ test). Moreover, presence of MSI+ lesions was correlated both with low Bcl-2 expression levels (*P*=0.02; *χ*^2^ test) and with p53 immunonegativity (*P*=0.04; *χ*^2^ test), whereas no significant association was found among MSI status and BSI (*P*=0.2; *χ*^2^ test).

### Univariate analysis for survival

**Figure 2:**
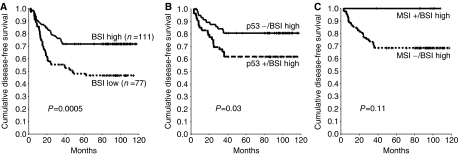
Kaplan–Meier DFS analysis for BAX (BSI) alone (**A**), for the p53/BAX high phenotype, subdivided into its two subtypes (p53−/BSI high or p53+/BSI high) (**B**), or for the MSI/BSI phenotype, subdivided into its two subtypes (MSI−/BSI high or MSI+/BSI high) (**C**).

**Figure 3:**
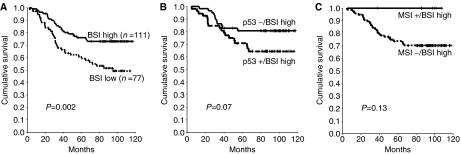
Kaplan–Meier OS analysis for BAX (BSI) alone (**A**), for the p53/BAX high phenotype, subdivided into its two subtypes (p53−/BSI high or p53+/BSI high) (**B**), or for the MSI/BSI phenotype, subdivided into its two subtypes (MSI−/BSI high or MSI+/BSI high) (**C**).

BAX expression revealed a significant impact on both DFS (Figure 2A; Kaplan–Meier curves for DFS) and OS (Figure 3A; Kaplan–Meier curves for OS), whereas the expression levels of neither Bcl-2 nor p53 or the MSI status were statistically significantly related to DFS or OS. Thus, 5-year DFS or OS in tumours with low and high BSI were 48.2 and 71.7%, respectively (*P*=0.0005, log-rank test), or 60.7 and 76.1%, respectively (*P*=0.002, log-rank test), whereas 5-year DFS or OS in patients with positive and negative Bcl-2 levels were 59.9 and 67.3%, respectively (*P*=0.7), or 69.3 and 71.3%, respectively (*P*=0.7). Similarly, 5-year DFS or OS in p53-positive and -negative cases were 57.0 and 67.3%, respectively (*P*=0.2), or 67.3 and 72.6%, respectively (*P*=0.2). Likewise, 5-year DFS or OS in MSI− and MSI+ individuals were 59.4 and 67.5%, respectively (*P*=0.51), or 68 and 81.3%, respectively (*P*=0.42).

The clinicopathological variables significantly associated with DFS and OS were lymph node category comparing pN1 with pN2-3 (5-year DFS 71.6 *vs* 49.3%; *P*=0.001; 5-year OS 80.2 *vs* 55.7%, *P*=0.0001), grade of differentiation when subdivided into G1/2 *vs* G3/4 (5-year DFS 66.8 *vs* 49.3%; *P*=0.005; 5-year OS 74.9 *vs* 55.6%, *P*=0.003) and treatment arm comparing arm A with arm B (5-year DFS 70.5 *vs* 53.2%; *P*=0.01; 5-year OS 76.7 *vs* 60.7%, *P*=0.02), whereas T category when subdivided into pT1/2 *vs* pT3/4 was only of borderline significance in correlation to DFS (5-year DFS 83.9 *vs* 59.7%, *P*=0.05).

Other clinicopathological parameters like age, sex and tumour site were not statistically significantly associated with DFS or OS (Table 2).

### Bivariate analysis of the BAX/Bcl-2−, the p53/BAX− and the BAX/MSI− phenotype

**Figure 4:**
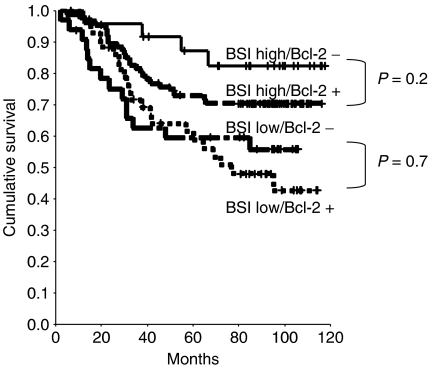
Kaplan–Meier OS analysis for the BAX/Bcl-2 group, subdivided in its four subsets (BSI high/Bcl-2−, BSI high/Bcl-2 +, BSI low/Bcl-2−, BSI low/Bcl-2+).

In view of the combined BAX/Bcl-2 phenotype with its four subtypes, 24 cases were BSI high/Bcl-2−, 87 were BSI high/Bcl-2+, 33 were BSI low/Bcl-2− and 44 were BSI low/Bcl-2+. The subset of BSI high/Bcl-2− cases was associated with the best prognosis (5-year DFS 82.6%; 5-year OS 87.1%); among patients with the BSI high/Bcl-2+ phenotype, outcome was associated with an intermediate prognosis (5-year DFS 68.8%; 5-year OS 73.1%) (Table 3), whereas patients harbouring the BSI low/Bcl-2− or + phenotype were found to have the worst prognosis (5-year DFS 56.8 or 41.4%; 5-year OS 61.4 or 59.5%) (Figure 4). Comparing patients with the BSI high/Bcl-2− or + phenotype as well as those with BSI low/Bcl-2− or + lesions showed no significant differences regarding 5-year DFS or 5-year OS (Table 3 and Figure 4).

With respect to the four subtypes of the p53/BAX phenotype, 58 lesions were identified as p53−/BSI high, 53 were p53+/BSI high, 36 were p53−/BSI low and 41 were p53+/BSI low. Subjects harbouring p53−/BSI high lesions exerted a favourable prognosis as compared to p53−/BSI low or p53+/BSI low cases (5-year DFS 80.7 *vs* 45.5 or 50.4%; 5-year OS 80.8 *vs* 58.5 or 62.6%), whereas patients with the p53+/BSI high phenotype were observed to have an intermediate-risk profile (5-year DFS 61.9%; 5-year OS 70.9%). Patients with BSI high/p53− tumours in comparison to patients with BSI high/p53+ lesions had an improved 5-year DFS (*P*=0.03) (Figure 2B; Kaplan–Meier curves for DFS) and a marginally improved 5-year OS (*P*=0.07) (Figure 3B; Kaplan–Meier curves for OS), whereas no such difference was noted among cases with BSI low/p53− or + tumours (Table 3).

Among 174 tumour specimens for which the MSI status was performed, the BSI high/MSI− phenotype was found in 97 samples, seven lesions were BSI high/MSI+, 61 were BSI low/MSI− and nine were BSI low/MSI+. Five-year DFS or OS in tumours with the BSI high/MSI+ phenotype were 100 and 100%, respectively, as compared with 68.6 or 73.7%, respectively, among those with the BSI high/MSI− phenotype (*P*=0.11 or *P*=0.13, respectively) (Figures 2C and 3C; Kaplan–Meier curves for DFS and OS). Five-year DFS or OS rates were 44.4 or 58.6% in the BSI low/MSI− group and 55.6 or 66.7% in the BSI low/MSI+ group (*P*=1.0 or *P*=0.9, respectively) (Table 3).

### Multivariate analysis

Multivariate analysis using Cox proportional hazards regression showed BAX expression to be a major independent prognostic factor for both DFS (Table 4) and OS (Table 5) (*P*=0.001 and *P*=0.003, respectively). Other independent negative predictive factors for both DFS and OS were the presence of two or more positive lymph nodes (*P*=0.005 and *P*<0.0001, respectively) and allocation to treatment arm B, respectively (*P*=0.008 and *P*=0.01, respectively). Other clinicopathological variables like age, gender, pT category and tumour location were not statistically significant independent markers in terms of DFS (Table 4) or OS (Table 5). Multivariate subgroup analysis including only subjects with BSI high tumours (*n*=111) and considering factors as covariates potentially to be correlated with disease recurrence and/or mortality like p53 status, pT category, pN category and treatment arm yielded p53 immunopositivity as the only independent marker for improved DFS (*P*=0.03), whereas it marginally failed to prove to be of significance in terms of OS (*P*=0.08). In this subset of patients, advanced lymph node category was a negative prognosticator of borderline significance for DFS and an independent factor for OS (*P*=0.066 and *P*=0.03, respectively) (data not shown).

## DISCUSSION

Recent reports by our group and others have suggested the proapoptotic BAX protein to act as a tumour suppressor in human malignancies (Zhan *et al*, 1994; Zhang *et al*, 2000; Schelwies *et al*, 2002; Klump *et al*, 2004) playing a key role in mediating the apoptotic programme in response to genotoxic stress (Theodorakis *et al*, 2002). For this reason, we examined, in a well-characterised, homogeneous collective of patients, whether BAX has a prognostic influence in stage III colon carcinoma treated by adjuvant chemotherapy. Additionally, we considered the interaction between proapoptotic BAX and its antiapoptotic counterpart Bcl-2 as well as its proposed transcriptional upstream regulator p53 in these series of experiments. Based on the observed prognostic value of these proteins by use of an immunohistochemical assay in colorectal cancer by our group and others (Sinicrope *et al*, 1995; Schelwies *et al*, 2002; Klump *et al*, 2004; Nehls *et al*, 2005), we followed this approach in the current study as well. High-frequency instability of microsatellites is present in about 15% of the cases of sporadic colorectal carcinoma, and it appears to be associated with different prognostic features as compared with tumours harbouring microsatellite stable or low-frequency microsatellite unstable lesions. Therefore, we screened 174 samples with enough DNA material for high-frequency MSI using the mononucleotide microsatellite marker BAT26. This marker alone was demonstrated to be sufficient to identify 97% of the MSI+ cases of 494 colorectal carcinoma samples (Loukola *et al*, 2001).

We found a high BSI or immunopositivity for Bcl-2 and p53 in 59, 70 and 50% of the cases, respectively. MSI+ lesions were detected in 9% of 174 evaluable patients. The observation in the present study that a high cytoplasmic BSI occurred with a lower frequency as compared to our previous rectal cancer trial (59 *vs* 68.5%) may be attributed to a slightly modified scoring system in our present series, in which we used the median BSI as cutoff value as described previously (Schelwies *et al*, 2002). The percentage of Bcl-2 and p53 immunopositivity (70 and 50%, respectively) is in good accordance with the results of a non-metastatic colon cancer study using both the same antibodies and the same scoring system as we did, thereby detecting cytoplasmic Bcl-2 and nuclear p53 immunopositivity in 66 and 48% of 110 cases, respectively (Sinicrope *et al*, 1995). The rate of MSI+ tumours observed in our study is in accordance with the data of Elsaleh *et al* (2001), who also found 9% MSI+ cases in a large series of stage III colorectal carcinoma using BAT26. In line with a previous study on colorectal cancer (Schelwies *et al*, 2002), we detected a reciprocal correlation between BAX expression and grading.

The main findings of our work were that high BSI was highly significantly associated with an improvement of both DFS and OS by multivariate analysis in 188 stage III colon carcinoma patients who had received cytotoxic therapy, whereas presence or absence of Bcl-2 or p53 expression alone was significantly correlated with clinical outcome.

Our data nicely confirm recent preclinical data showing that BAX is of crucial significance for the induction of apoptosis by 5-FU in colon cancer cell lines (Theodorakis *et al*, 2002). Moreover, a positive prognostic relevance of high BAX expression in 41 colorectal cancer patients who underwent curatively intended resection of liver metastases has been reported – since a substantial proportion of these patients (71%) had received 5-FU-based cytotoxic therapy after complete surgical resection of their lesions, we feel that these data are in good concordance with ours (Sturm *et al*, 1999). Furthermore, we reported in our initial studies on 92 rectal cancer patients, all treated by neoadjuvant radiotherapy concurrent to surgery, that a high BSI was independently linked to an improved rate of disease recurrence underlining the potential predictive value of BAX in the setting of genotoxic therapy in colorectal cancer as well (Nehls *et al*, 2005).

Although BAX may be a central effector of its proposed transcriptional regulator, the tumour suppressor gene p53 (Miyashita and Reed, 1995), we found, consistent with the results of others (Watanabe *et al*, 2001), no significant correlation between p53 protein expression alone and outcome in our series. However, according to another trial, p53 was found to predict clinical outcome in colorectal cancer patients treated by adjuvant 5-FU-based treatment (Ahnen *et al*, 1998). Hypothetically, these contradictory results may be explained by differential expressions of BAX, which was supposed to act as a central downstream effector of p53 (Miyashita and Reed, 1995). Accordingly, in the present trial, subgroup analysis showed that patients with p53−/BSI high tumours were less likely to experience relapse or death than those harbouring p53+/BSI high lesions (5-year DFS 80.7 *vs* 61.9 months, *P*=0.03; 5-year OS 80.8 *vs* 70.9 months, *P*=0.07, respectively), suggesting a positive association between an intact p53/BAX pathway and clinical outcome. Thus, the proapoptotic effect of BAX, positively influencing clinical behaviour, may even be more corroborated in patients with p53-immunonegative lesions. A similar correlation was found in a recent report on metastatic colorectal cancer demonstrating a clear survival advantage for patients with an intact p53/BAX pathway (p53 wild type/BAX high) as well (Sturm *et al*, 1999).

Therefore, BAX might constitute a critical element probably mediating cell susceptibility to undergo apoptotic cell death, which additionally might depend on the p53 expression status in the context of genotoxic stress in colon cancer.

We also evaluated the prognostic role of antiapoptotic Bcl-2, the so-called counteracting twin of BAX (Korsmeyer, 1999). However, in contrast to BAX, Bcl-2 expression alone was not observed to be prognostically useful in stage III colon cancer, which is in line with previous reports on the prognostic significance of Bcl-2 in adjuvantly treated non-metastatic colorectal cancer (Tollenaar *et al*, 1998; Garrity *et al*, 2004). In addition, combined analysis of the BAX/Bcl-2 phenotypes did not confer additional significant prognostic information over BAX alone. Thus, a high BSI combined with either negative or positive Bcl-2 levels was associated with both improved DFS and OS as compared to tumours that showed a low BSI combined with either Bcl-2-negative or -positive samples. Nevertheless, in contrast to BSI low/Bcl-2− or + individuals showing essentially no difference for long-term survival (5-year OS 61.4 *vs* 59.5%), patients with the BSI high/Bcl-2− phenotype, as depicted in Figure 4, had an excellent outcome that was moderately better as compared to BSI high/Bcl-2+ cases (5-year OS 87.1 *vs* 73.1%), but the shift of the BAX to Bcl-2 ratio in favour of the BSI high/Bcl-2− phenotype did not translate into a statistically significant difference for clinical outcome in our series of cytotoxically treated stage III colon cancer (OS, *P*=0.2). Consequently, the balance of the BAX to Bcl-2 ratio may *in vivo* functionally be predominated by the expression status of BAX, which is consistent with the recent observation that the proapoptotic activity of the multidomain protein BAX might function independently of the Bcl-2 status (Knudson and Korsmeyer, 1997). Furthermore, it has recently been demonstrated that members of the Bcl-2 family other than Bcl-2 also may counter BAX by forming heterodimers, thereby inhibiting the apoptotic programme (Sedlak *et al*, 1995). Thus, Bcl-2 expression possibly may not be of central prognostic significance in cytotoxically treated stage III colon carcinoma.

Our observation that patients with MSI+ tumours did not have a statistically significant better outcome as compared to those with MSI− neoplasms is in line with the recent results of Ribic *et al* (2003), demonstrating that patients who received adjuvant 5-FU-based chemotherapy and with MSI+ lesions had no better outcome than those with MSI− neoplasms. However, others found a significant survival benefit in MSI+ cases who received adjuvant 5-FU-based chemotherapy (Elsaleh *et al*, 2001). These conflicting results may be explained by preclinical studies demonstrating that MSI+ tumours were less responsive to 5-FU as compared to MSI− lesions (Carethers *et al*, 1999), which has been attributed to the observation that mismatch repair proteins may be essential to incorporate 5-FU into tumour DNA (Tajima *et al*, 2004), whereas others found that MSI+ tumours may harbour an intrinsic favourable prognosis (Popat *et al*, 2005).

Notably, bivariate subgroup analysis showed a nonsignificant trend for survival benefit for patients with the BSI high/MSI+ phenotype as compared to those with the BSI high/MSI− phenotype. Furthermore, in all patients with the BSI high/MSI+ phenotype, neither was there recurrence of disease nor any disease-related death. Possibly, this subgroup of patients may harbour an inherent favourable prognosis. However, these observations should be interpreted with caution, given that only nine patients had BSI high/MSI+ lesions. Therefore, prospective studies on larger series may address this issue.

In conclusion, this study for the first time indicates a positive predictive role of high BSI in the outcome of adjuvant chemotherapy in stage III colon cancer. Thus, preclinical data showing BAX expression to be of vital impact for 5-FU-induced apoptosis in colon cancer cell lines are supported by the data presented here. However, monogenic analysis of both the proposed upstream regulator of BAX, p53, and its antiapoptotic relative, Bcl-2, did not provide additional prognostic information either, whereas bivariate analysis of the p53/BAX phenotype and the BSI high/MSI+ phenotype possibly may add further prognostic evidence.

Accordingly, it might be speculated that BAX enhances chemotherapy-induced apoptosis by a pathway that does not depend fundamentally on the BAX to Bcl-2 balance, but may be influenced by intact p53 or by the MSI status. Therefore, prospective studies evaluating whether the status of BAX protein alone or in combination with p53 or MSI may contribute to the identification of stage III patients who are benefited from adjuvant chemotherapy are strongly suggested by these data.

## Figures and Tables

**Table 1A:** Clinicopathological parameters in relation to p53, BAX and Bcl-2 protein expression (*n*=188)

		**BSI (no. (%))**			**Bcl-2 (no. (%))**			**p53 (no. (%))**		
	***n***	**High**	**Low**	***P***	**High**	**Low**	***P***	**Positive**	**Negative**	***P***
*Sex*										
Male	79	44 (55.7)	35 (44.3)	0.43	52 (65.8)	27 (44.2)	0.33	42 (53.2)	37 (46.8)	0.46
Female	109	67 (61.5)	42 (38.5)		79 (72.5)	30 (27.5)		52 (47.7)	57 (52.3)	
										
*Age, years*										
⩽62	98	63 (64.3)	35 (35.7)	0.13	68 (69.4)	30 (30.6)	0.93	52 (53.1)	46 (46.9)	0.38
>62	90	48 (53.3)	42 (46.7)		63 (70.0)	27 (30.0)		42 (46.7)	48 (53.3)	
										
*T category, pathologic*										
T2	19	11 (57.9)	8 (42.1)	0.92	15 (78.9)	4 (21.1)	0.35	10 (52.6)	9 (47.4)	0.81
T3–4	169	100 (59.2)	69 (40.8)		116 (68.6)	53 (31.4)		84 (49.7)	85 (50.3)	
										
*N category, pathologic*										
N1	107	67 (62.6)	40 (37.4)	0.25	75 (70.1)	32 (29.9)	0.89	49 (45.8)	58 (54.2)	0.19
N2–3	81	44 (54.3)	37 (45.7)		56 (69.1)	25 (30.9)		45 (55.6)	36 (44.4)	
										
*Differentiation*										
G1–2	138	89 (64.5)	49 (35.5)	**0.01**	97 (70.3)	41 (29.7)	0.76	69 (50.0)	69 (50.0)	1
G3–4	50	22 (44.0)	28 (56.0)		34 (68.0)	16 (32.0)		25 (50.0)	25 (50.0)	
										
*Location*										
Right	93	51 (54.8)	42 (45.2)	0.25	58 (62.4)	35 (37.6)	**0.03**	43 (46.2)	50 (53.8)	0.31
Left	95	60 (63.2)	35 (36.8)		73 (76.8)	22 (23.2)		51 (53.7)	44 (46.3)	
										
*Treatment arm*										
Arm A (5-FU/LV)	95	56 (58.9)	39 (41.1)	0.98	69 (72.6)	26 (27.4)	0.37	44 (46.3)	51 (53.7)	0.31
Arm B (5-FU/LEV)	93	55 (59.1)	38 (40.9)		62 (66.7)	31 (33.3)		50 (53.8)	43 (46.2)	

G1, well; G2, moderately; G3, poorly; G4, anaplastic. Bold values signify *P*<0.05.

**Table 1B:** Clinicopathological parameters in relation to microsatellite status (*n*=174)

	***n***	**MSI− (no. (%))**	**MSI+ (no. (%))**	***P***
*Sex*				
Male	76	71 (93)	5 (7)	0.29
Female	98	87 (89)	11 (11)	
				
*Age, years*				
⩽62	93	88 (95)	5 (5)	0.06
> 62	81	70 (86)	11 (14)	
				
*T category, pathologic*				
T2	18	18 (100)	0 (0)	0.38
T3–4	156	140 (90)	16 (10)	
				
*N category, pathologic*				
N1	99	92 (93)	7 (7)	0.27
N2–3	75	66 (88)	9 (12)	
				
*Differentiation*				
G1–2	125	115 (92)	10 (8)	0.38
G3–4	49	43 (88)	6 (12)	
				
*Location*				
Right	87	71 (82)	16 (18)	<**0.0001**
Left	87	87 (100)	0 (0)	
				
*Treatment arm*				
Arm A (5-FU/LV)	90	84 (93)	6 (7)	0.23
Arm B (5-FU/LEV)	84	74 (88)	10 (12)	

G1, well; G2, moderately; G3, poorly; G4, anaplastic. Bold values signify *P*<0.05.

**Table 2:** Analysis of DFS and OS in relation to clinicopathological parameters, p53, BAX and Bcl-2 protein expression and MSI status

	***n***	**5-year DFS (%)**	***P***	**5-year OS (%)**	***P***
*Sex*					
Male	79	57.1	0.2	68.8	0.3
Female	109	65.7		70.7	
					
*Age, years*					
⩽62	98	64.7	0.6	72.7	0.4
>62	90	59.4		66.8	
					
*T category, pathologic*					
T2	19	83.9	0.05	83.9	0.1
T3–4	169	59.7		68.3	
					
*N category, pathologic*					
N1	107	71.6	**0.001**	80.2	**0.0001**
N2–3	81	49.3		55.7	
					
*Differentiation*					
G1–2	138	66.8	**0.005**	74.9	**0.003**
G3–4	50	49.3		55.6	
					
*Location*					
Right	93	66.9	0.1	74.2	0.3
Left	95	57.1		65.5	
					
Treatment arm					
Arm A (5-FU/LV)	95	70.5	**0.01**	76.7	**0.02**
Arm B (5-FU/LEV)	93	53.2		62.4	
					
BAX staining index					
High	111	71.7	**0.0005**	76.1	**0.002**
Low	77	48.2		60.7	
					
Bcl-2 expression					
Positive	131	59.9	0.7	69.3	0.7
Negative	57	67.5		71.3	
					
p53 expression					
Positive	94	57	0.2	67.3	0.2
Negative	94	67.3		72.6	
					
MSI status					
MSI−	158	59.4	0.5	68	0.4
MSI+	16	67.5		81.3	

Kaplan–Meier curves compared by log-rank test.

DFS=disease-free survival; MSI=microsatellite instability; OS=overall survival. Bold values signify *P*<0.05.

**Table 3:** Bivariate subgroup analysis of patients with high or low BSI lesions combined with either Bcl-2−/+ or p53−/+ or MSI−/+ samples in relation to 5-year DFS and 5-year OS (log-rank test)

	***n***	**5-year DFS (%)**	***P***	**5-year OS (%)**	***P***
BSI high/Bcl-2−	24	82.6	0.2	87.1	0.2
BSI high/Bcl-2+	87	68.8		73.1	
BSI low/Bcl-2−	33	56.8	0.7	59.5	0.7
BSI low/Bcl-2+	44	41.4		61.4	
BSI high/p53−	58	80.7	**0.03**	80.8	0.07
BSI high/p53+	53	61.9		70.9	
BSI low/p53−	36	45.5	0.7	58.5	0.8
BSI low/p53+	41	50.4		62.6	
BSI high/MSI−	97	68.6	0.11	73.7	0.13
BSI high/MSI+	7	100		100	
					
BSI low/MSI−	61	44.4	1	58.6	0.9
BSI low/MSI+	9	55.6		66.7	

BSI=BAX staining index; DFS=disease-free survival; 5-FU=5-fluorouracil; LEV=levamisole; MSI=microsatellite instability; OS=overall survival. Bold values signify *P*<0.05.

**Table 4:** Multivariate analysis of clinicopathological and genetic parameters for DFS (Cox regression hazard analysis)

	**DFS**		
**Prognostic factor**	***P*-values**	**Hazard ratio**	**95% CI**
Step 1			
*pT category*			
T3/4	0.11	2.6	0.8–8.3
			
*pN category*			
N2/3	**0.008**	1.9	1.2–3.1
			
*Differentiation*			
G3/4	0.22	1.4	0.8–2.3
			
Treatment arm			
Arm B (5-FU/LEV)	**0.01**	1.9	1.2–3.0
			
*BSI*			
Low	**0.001**	2.2	1.4–3.6
			
Step 2			
*pT category*			
T3/T4	0.09	2.7	0.9–8.7
			
*pN category*			
N2/3	**0.005**	2	1.2–3.2
			
*BSI*			
Low	**0.001**	2.3	1.4–3.7
			
*Treatment arm*			
Arm B (5-FU/LEV)	**0.008**	1.9	1.2–3.1

CI=confidence interval; DFS=disease-free survival; 5-FU=5-fluorouracil; LEV=levamisole. Bold values signify *P*<0.05.

**Table 5:** Multivariate analysis of clinicopathological and genetic parameters for OS (Cox regression hazard analysis)

	**OS**		
**Prognostic factor**	***P*-values**	**Hazard ratio**	**95% CI**
Step 1			
*pT category*			
T3/4	0.27	2	0.6–6.3
			
*pN category*			
N2/3	**0.001**	2.3	1.4–3.8
			
*Differentiation*			
G3/4	0.18	1.4	0.9–2.4
			
*Treatment arm*			
Arm B (5-FU/LEV)	**0.02**	1.8	1.1–3.8
			
*BSI*			
Low	**0.004**	2.1	1.3–3.4
			
Step 3			
*pN category*			
N2/3	**<0.0001**	2.5	1.5–4.2
			
*BSI*			
Low	**0.003**	2.1	1.3–3.5
			
*Treatment arm*			
Arm B (5-FU/LEV)	**0.01**	1.9	1.1–3.1

CI=confidence interval; 5-FU=5-fluorouracil; LEV=levamisole; OS=overall survival. Bold values signify *P*<0.05.
